# Safety of Rituximab biosimilar (Riximyo®) following a single switch from the reference product in patients with Non-Hodgkin’s lymphoma: a retrospective study

**DOI:** 10.1007/s00277-024-05981-9

**Published:** 2024-09-09

**Authors:** Nina K Song, Hala Musa, Michael Soriano, David E Hibbs, Iqbal Ramzan, Jennifer A Ong

**Affiliations:** 1 School of Pharmacy, University of Sydney, Sydney, NSW 2006 Australia; 2 Pharmacy Department, Chris O’Brien Lifehouse, Camperdown, NSW 2006 Australia

**Keywords:** Biosimilars, Switching, Rituximab, GP2013, Safety

## Abstract

Unlike small molecule drugs and generic products, the active component of biologics and biosimilars are not identical chemical entities. Despite bioequivalence, there is limited evidence in clinical practice (i.e. Phase IV post-marketing surveillance) regarding the safety of biosimilar rituximab and even less so for “switching therapy” with respect to safety. Drug substitution by switching aims to realise cost savings by changing therapy involving a reference (biologic) product to a biosimilar. A retrospective analysis of safety outcomes including treatment-emergent adverse effects (TEAEs), rates of death and discontinuation of therapy, for all patients that received switching therapy (from reference to biosimilar rituximab, *n* = 33) was compared to patients who did not did not switch therapy (received biosimilar rituximab only, *n* = 18) at an Australian metropolitan cancer centre, over a six-month period. There was no statistical significant differences for any safety outcomes examined. Switching therapy for patients receiving rituximab does not lead to poorer safety outcomes.

## Introduction

Rituximab is a chimeric monoclonal antibody (mAb) used to treat several types of non-Hodgkin’s lymphoma (NHL) in combination with traditional therapy. NHL is the sixth and seventh most common cancer in men and women, making up 4% of all new cancers in the United States, with the most standard therapy consisting of anti-CD20 immuno- or chemotherapy [1]. Despite increasing trends of NHL worldwide, death and disability-adjusted life years (DALY) have been decreasing, especially in high and middle-high sociodemographic index areas [2]. Rituximab targets the B-cell antigen CD20, a cell surface antigen related to a variety of B-cell proliferation mechanisms including the regulation of intracellular calcium, cell cycle, and apoptosis [3]. Rituximab is commonly used with CHOP therapy, consisting of cyclophosphamide, doxorubicin, vincristine, and prednisone, and has dramatically improved progression-free survival and overall survival with 74.3% survival at 6 years for the most common NHL subtype, diffuse large B-cell lymphoma (DLBCL), without an increase in adverse events compared to CHOP only therapy [3–5]. Significant improvement in survival resulted in rituximab being included in the WHO Model List of Essential Medicines for cancer in 2015, however access has been an issue, especially in lower income countries as rituximab is much more expensive compared to traditional CHOP therapy [2].

In Australia, the Pharmaceutical Benefits Scheme (PBS) first approved the use of rituximab in relapse or refractory low-grade or follicular CD20-positive, B-cell NHL on 6 October 1998, and for use in CD20-positive, DLBCL in combination with chemotherapy in April 2004 [6]. However, despite traditional CHOP therapy being cost-friendly and accessible to all patients, rituximab remains expensive and causes a significant economic burden to the healthcare system. In the 2018 PBS report, rituximab was the 17th most expensive PBS drug, costing $124,680,015 per annum [7]. On 30 November 2017, the first rituximab biosimilar Riximyo^®^ (GP2013) manufactured by Sandoz was introduced to Australia, however PBS subsidy was not approved until much later on 1 October 2019 [8]. There have since been two more biosimilars which have been made available in Australia: Truxima^®^ (Celltrion) since 16 April 2018 (subsidised since 1 January 2020), and Ruxience^®^ (Pfizer) since 3 March 2021 (not subsidised) [8]. Through the introduction of biosimilars, over a 25% price reduction on the approved ex-manufacturer price has been effected in Australia, and federal government expenditure for rituximab was reduced by approximately $40 M annually in the first year of biosimilar introduction (2019/2020) and over $18 M AUD in the following year (2020/1) [7]. This aligns with the predicted reduction of $43 M in the first year of biosimilar implementation by Gleeson et al. 2019, followed by a further reduction of $23 M in the second year [9]. Furthermore, the PBS has since ceased subsidy for intravenous (IV) and subcutaneous (SC) formulations of rituximab originator MabThera^®^ which has subsequently reduced patient access to the medication [10]. Thus, confidence in prescribing and using biosimilar rituximab in all eligible patients to maintain cost-effectiveness and access to therapy is essential, especially as the PBS has now recognised Riximyo^®^ and Truxima^®^ as ‘substitutable biosimilars’, permitting substitution by pharmacists.

Although there is evidence to suggest that switching to biosimilars such as infliximab [11, 12] and epoetins [13] do not lead to safety concerns, perceived issues with rituximab biosimilar still exist due to the prescriber and patients’ belief in the comparability between originator and biosimilar rituximab [14]. While prescribers have a positive outlook on biosimilars, there is still limited prescribing [15]. This is because biosimilar rituximab, like all other biosimilars, does not share exactly the same molecular structure as the originator rituximab. Approved biosimilars are required to show significant similarity in molecular structure, efficacy, and safety, whereby small changes in the clinically inactive components are tolerated [16]. The clinically inactive components are less of a concern in patients who are initiated on a biosimilar but may present as potential issues in patients who were previously treated with the originator rituximab, due to immunogenicity – the formation of anti-drug antibodies which reduce the efficacy of the treatment. These patient groups are known as ‘switching patients’ and form a large proportion of the population receiving this treatment in clinical practice. Riximyo^®^ has been proven to be clinically equivalent to MabThera^®^ the reference product in a Phase III clinical trial (ASSIST-FL) by Jurczak et al. 2017 in combination with CVP (cyclophosphamide, vincristine, and prednisone) for follicular lymphoma [17].

To address these arising concerns regarding switching patients in clinical practice, Urru et al. 2021 conducted a prospective observational study in Italy to evaluate the safety of Truxima^®^ vs. Rixathon^®^ vs. MabThera^®^ in patients with NHL and Chronic Lymphocytic Leukemia [18]. The switch was conducted between biosimilars only (and not from the reference product) and presented no significant safety signals associated with the act of switching or the use of between biosimilars, in alignment with previously available evidence from the Phase III clinical trials [17]. However, evidence to support the practice of switching patient who are already established on MabThera^®^ is still scarce, hence this was investigated through a retrospective analysis of medical records from an Australian metropolitan cancer centre in this current study.

### Objectives

The primary objective of this study was to evaluate the safety profile of rituximab biosimilar GP2013 following a single switch from the reference product (R) compared to patients who were administered the biosimilar only.

## Methods

This study was approved by the Sydney Local Health District Human Research Ethics Committee (LH21.042) in accordance with the National Health Medical Research Council Statement on Ethical Conduct in Human Research (2007).

### Study design

This study was a retrospective single-centre analysis conducted at a cancer centre in Sydney, New South Wales, Australia. Data were collected from electronic medical records to assess the frequency of adverse events, immunogenicity and discontinuation of therapy rates for patients commencing or switching to GP2013 over a six-month period (between 18/11/19 to 18/5/20). There was a follow-up period of 12 months in both arms.

### Participants

Eligibility criteria included the following: any patient who was administered at least one dose of biosimilar rituximab GP2013; aged 18 years or older with histologically confirmed lymphoma; and had adequate cardiac function (LVEF ≥ 45%). All switching patients were informed about changing over to a biosimilar beforehand.

### Study procedures

Data for patients who received GP2013 between 18 November 2019 and 18 May 2020 were extracted from an electronic medical records database available on-site. Two investigators (HM, MS) collected and de-identified data on-site and recorded outcomes on a predetermined data collection tool created in REDCap [19, 20]. Two investigators (NS, JO) conducted statistical analysis using the software packing SPSS [21].

### Adverse events

Data collected included demographic data and any treatment-emergent adverse effects (TEAEs) which are known and have been previously reported such as: anaemia, arthralgia, asthenia, cardiac failure, constipation, cough, hypersensitivity, infection/infestation, infusion reaction, nausea and vomiting, neutropenia, pulmonary nephropathy, pulmonary toxicity, cardiotoxicity and urinary tract infection (UTI). Immunogenicity was assessed through the formation of anti-drug antibodies (ADAs) and neutralising antibodies (Nabs) in patients reported.

All adverse event grades are defined by the Common Terminology Criteria for Adverse Events (CTCAE) [22]. Grade 1 was defined as asymptomatic or mild symptoms, Grade 2 as moderate/minimal/local symptoms, and Grade 3 as severe but not life-threatening. Grade 4 corresponded with life-threatening consequences where urgent intervention/discontinuation was required. Grade 5 was defined as death related to adverse events. Where medical records did not specify the grade or severity of the reaction, grading was omitted. Any reason for treatment discontinuation and death prior to the end of the treatment regimen was also noted. Previous treatment with other anti-tumour therapy (i.e. whether or not the patient was treatment-naïve) was also noted.

### Statistical analyses

Statistical analysis was conducted according to a predetermined protocol on all eligible patients who presented with any TEAE, cardiotoxicity, immunogenicity, or any reason for discontinuation). Based on the predetermined statistical analysis plan, primary safety analysis of frequency data was analysed and compiled for all patients. Results from patients who have only received biosimilar GP2013 were compared against patients who switched from R, MabThera^®^. Descriptive statistical analysis was carried out for demographic data including age, gender, NHL pathological type, medication history (particularly previous exposure to any other biologic treatment: neoadjuvant, adjuvant, maintenance), and ECOG performance status. To test for independence the Mann-Whitney U test and Chi-squared test (or Fisher’s Exact test) were undertaken for quantitative and categorical data, respectively. All statistical analyses were conducted using the software SPSS and *p*-values ≤.05 was considered to be statistically significant.

## Results

**Fig. 1:**
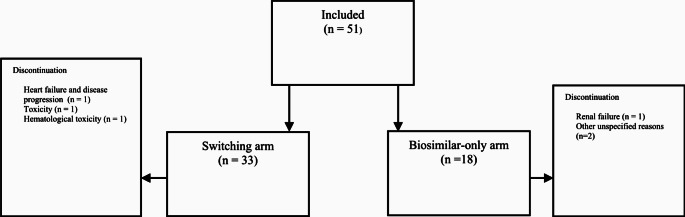
Diagram showing study arms

A total of 51 patients (biosimilar-only arm: *n* = 18, switching arm: *n* = 33) were included (Fig. 1). Key demographics are summarised in Table 1. No patients died during the study period, and a total of *six* patients discontinued treatment before completion [*n* = 3 (17%) and *n* = 3 (9%) in biosimilar-only and switching arms, respectively]. Reasons for discontinuation included renal failure attributed to dehydration (*n* = 1) and unspecified reasons (*n* = 2) for the biosimilar-only arm. For the switching arm, the reasons were exacerbated heart failure and disease progression (*n* = 1), toxicity and reduced QOL with chemotherapy (*n* = 1), and haematological toxicity (*n* = 1). There appeared to be no increase in odds of discontinuation for any reason associated with the switching arm [odds ratio (OR): 0.8, 95% CI: 0.1–5.3, *p*-value = 0.82] (Table 2). Immunogenicity, namely ADAs and Nabs formed in patients nor deaths were not reported in any of the records.

**Table 1:** Participant demographics

Subgroup	GP2013/GP2013 (*n* = 18)	R/GP2013 (*n* = 33)	*p*-value
Age (years), median (IQR)^a^	74 (52–79)	69 (59–76)	0.615
Gender, male, n (%)^*b*^	10 (56)	20 (61)	0.772
Pathological type, n (%)^*c*^ CLL DLBCL Follicular lymphoma Hodgkin’s lymphoma MALT lymphoma Marginal zone lymphoma Waldenström’s macroglobulinemia Uncertain subtypes of B cell lymphoma	0 (0.0) 6 (33.3) 1 (5.6) 1 (5.6) 0 (0.0) 0 (0.0) 1 (5.6) 9 (50.0)	2 (6.1) 9 (27.3) 4 (12.1) 0 (0.0) 2 (6.1) 1 (3.0) 2 (6.1) 13 (39.4)	0.803
ECOG Performance Status, median (IQR)^a^	1.0 (1.0–2.0)	1.5 (1.0–2.0)	0.424
Cycle no. of first administration of biosimilar (cycle), median (IQR) ^*a*^	1.0 (1.0–1.0)	3.0 (2.0–4.0)	0.001
Treatment naïve, n (%)^*b*^	13 (72)	27 (82)	0.634

CLL: Chronic lymphocytic leukaemia, DLBCL: Diffuse large B cell lymphoma, ECOG: Eastern Cooperative Oncology Group

^*a.*^ *Mann-Whitney U test*

^*b.*^ *Chi-squared test*

^*c.*^ *Fisher’s Exact test*

**Table 2:** Number of patients who died or discontinued for any reason

	GP2013/GP2013, *n* (%)	*R*/GP2013, *n* (%)	OR (95% CI)	*p*-value
Discontinued for any other reason	3 (17)	6 (9)	0.8 (0.1–5.3)	0.82

### Treatment-Emergent Adverse Events (TEAEs)

There were five TEAEs in the biosimilar-only group compared to 24 events in the switching arm (Table 3).

TEAEs which were experienced most frequently in the switching arm were low-grade nausea and vomiting (*n* = 10, 30.3%), followed by constipation (*n* = 4, 12.1%), then anaemia (*n* = 2, 6.1%), cardiac failure (*n* = 2, 6.1%), and infection/infestation (*n* = 2, 6.1%). Similarly, the TEAEs that were experienced most frequently in the biosimilar-only arm were nausea and vomiting (*n* = 3, 16.7%), followed by constipation (*n* = 1, 5.6%) and anaemia (*n* = 1, 5.6%). There was no statistically significant increase in any TEAE in the switching arm compared to the biosimilar-only arm. Where reported, severity of TEAEs were mild or moderate and did not exceed Grade 2.

**Table 3:** Summary of TEAEs in biosimilar-only and switched arms

TEAEs, *n* (%)	GP2013/GP2013 (*n* = 18)	R/GP2013 (*n* = 33)	*p*-value
Anaemia	1 (5.6)	2 (6.1)	1.00
Arthralgia	0 (0.0)	1 (3.0)	1.00
Cardiac failure	0 (0.0)	2 (6.1)	0.534
Constipation	1 (5.6)	4 (12.1)	0.645
Cough	0 (0.0)	1 (3.0)	1.00
Infection/infestation	0 (0.0)	2 (6.1)	0.534
Nausea and vomiting	3 (16.7)	10 (30.3)	0.336
Neutropenia	0 (0.0)	1 (3.0)	1.00
Pulmonary nephropathy	0 (0.0)	1 (3.0)	1.00

^*a*^Fisher’s Exact test

## Discussion

This retrospective study suggests comparable safety of rituximab biosimilar, Riximyo^®^ in patients who receive this agent at treatment commencement (biosimilar-only arm) and those who were switched from the originator R MabThera^®^ (switching arm). The limitations of this work which includes a retrospective study design, small and heterogenous sample obtained from a single centre, and a relatively short follow-up period (12 months), leads us to report the lack of statistically significant difference in TEAEs detected between the two arms cautiously. Nonetheless, our findings align with previous studies including a randomised clinical trial and another observational study (with median follow-up periods ranging from 10.5 to 27 months), which found single switches to be well-tolerated with similar rates of adverse effects (in terms of both seriousness and frequency) [18, 23].

Overall, there was no meaningful difference in the baseline demographics despite treatment cycle number at commencement of GP2013. Pathological type DLBCL and the R-CHOP treatment, standard therapy for NHL consisting of rituximab with cyclophosphamide, doxorubicin hydrochloride, vincristine and prednisone was the most commonly prescribed in both arms. Most patients were treatment-naïve and had not previously received therapy for lymphoma whilst the remainder had previously experienced disease relapse. Findings showed no difference in the incidence of TEAEs, death or treatment discontinuation for any reason in the switching arm. However, these findings must be interpreted with caution due to the small sample size. Nonetheless, this work complements previous research which has established the comparability in both safety and efficacy by direct head-to-head comparison of biosimilar-only against originator-only therapy in treatment-naïve patients. A systematic review by Lee et al. (2019) reported no differences in terms of immunogenicity, safety, or efficacy for treatment-naïve patients in an oncological setting [24]. In the Phase III clinical trial ASSIST-FL, infusion reaction and nausea and vomiting were the main adverse events for both the originator and the biosimilar. While no infusion-related reactions were detected for either arm in the present study, the incidence of TEAEs in the biosimilar-only arm in this study was comparable to that in the ASSIST-FL trial (16.7% vs. 11%, respectively) [17]. Generalisability may be limited to people who are relatively well with favourable performance status and receiving rituximab as part of the R-CHOP regimen, as different combinations of drugs in the regimen will inherently contribute to a varied side effect profiles. For example, the RICE regimen which includes highly emetogenic medications including ifosfamide and carboplatin (in addition to rituximab and etoposide) and lacks an antiemetic corticosteroid, may result in greater incidences and severity of nausea and vomiting.

Despite the positive reception of biosimilars by physicians, further evidence with a larger sample size would be beneficial to increase prescribing confidence [15], and indeed generic substitution by pharmacists. Similarly, the collection of efficacy data in this setting was considered premature as the full switch to Riximyo^®^ began in November 2019, thus a long-term biosimilar efficacy study would be beneficial to boost clinical confidence, as results are still scarce in oncological and clinical settings outside clinical trials [25]. Furthermore, data for the formation of ADAs and Nabs should be recorded in clinical settings to investigate the impact of switching on the formation of antibodies and immunogenicity.

## Conclusion

This retrospective study suggests that the safety of rituximab biosimilar GP2013 in switching patients is comparable to that in patients who received the biosimilar only throughout the entire treatment. Results showed that there was no significant difference in the incidence of adverse events, however, a larger study should be undertaken to produce conclusive evidence. Immunogenicity as a result of switching is still unknown and clinical settings are encouraged to purposefully note incidences of this for further analysis.

## Data Availability

No datasets were generated or analysed during the current study.
